# Physiological Responses of Two Contrasting Kiwifruit (*Actinidia* spp.) Rootstocks against Waterlogging Stress

**DOI:** 10.3390/plants10122586

**Published:** 2021-11-25

**Authors:** Zhi Li, Danfeng Bai, Yunpeng Zhong, Muhammad Abid, Xiujuan Qi, Chungen Hu, Jinbao Fang

**Affiliations:** 1 Key Laboratory for Fruit Tree Growth, Development and Quality Control, Zhengzhou Fruit Research Institute, Chinese Academy of Agricultural Sciences, Zhengzhou 450009, China; lizhizzgss21@163.com (Z.L.); 82101199221@caas.cn (D.B.); zhongyunpeng@caas.cn (Y.Z.); muhammadabid@lsbg.cn (M.A.); qixiujuan@caas.cn (X.Q.); 2 Key Laboratory of Horticultural Plant Biology, College of Horticulture & Forestry Sciences of Huazhong Agricultural University, Wuhan 430070, China

**Keywords:** *Actinidia valvata*, adventitious root, amino acid, photosynthesis, sucrose, waterlogging

## Abstract

Rootstocks from *Actinidia valvata* are much more tolerant to waterlogging stress than those from *Actinidia deliciosa*, which are commonly used in kiwifruit production. To date, the tolerance mechanism of *A. valvata* rootstocks’ adaptation to waterlogging stress has not been well explored. In this study, the responses of KR5 (*A. valvata*) and ‘Hayward’ (*A. deliciosa*) to waterlogging stress were compared. Results showed that KR5 plants performed much better than ‘Hayward’ during waterlogging by exhibiting higher net photosynthetic rates in leaves, more rapid formation of adventitious roots at the base of stems, and less severe damage to the main root system. In addition to morphological adaptations, metabolic responses of roots including sufficient sucrose reserves, modulated adjustment of fermentative enzymes, avoidance of excess lactic acid and ethanol accumulation, and promoted accumulation of total amino acids all possibly rendered KR5 plants more tolerant to waterlogging stress compared to ‘Hayward’ plants. Lysine contents of roots under waterlogging stress were increased in ‘Hayward’ and decreased in KR5 compared with their corresponding controls. Overall, our results revealed the morphological and metabolic adaptations of two kiwifruit rootstocks to waterlogging stress, which may be responsible for their genotypic difference in waterlogging tolerance.

## 1. Introduction

Some orchards occasionally experience waterlogging stress owing to intense rainfall or over-irrigation [1]. For fruit trees, the performance of aboveground plant parts under waterlogging stress is associated with potential economic loss. Plants usually respond to waterlogging stress by closing the stomata of leaves, thereby compromising photosynthesis [2,3]. Leaves also suffer oxidative damage under waterlogging stress. Malondialdehyde (MDA) accumulation can cause lipid peroxidation and consequently damage the membrane [4,5]. Leaves are an important source of nutrients, and the degree of leaf damage under waterlogging stress reflects the tolerance of the whole plant. Under flooding conditions, the stem bases of plants are frequently immersed in water together with the roots. Lenticel hypertrophy and formation of adventitious roots at the stem base are important morphological adaptations in cucumber, maize, and tomato plants [6,7,8].

A decline in the oxygen concentration around the root rhizosphere greatly hinders mitochondrial respiration [9]. To manage this energy crisis, glycolysis and fermentation are typically activated in roots under waterlogging stress. Catabolism of soluble sugars or starch provides substrates for glycolysis. Sugar availability in hypoxic tissues is crucial for cell viability and survival [10,11]. For flood-sensitive plants, survival is threatened once carbohydrate substrates are exhausted [12]. Ethanolic fermentation provides nicotinamide adenine dinucleotide (NAD^+^) to maintain glycolysis. Pyruvate decarboxylase (PDC) and alcohol dehydrogenase (ADH) are two important fermentative enzymes that can reflect the tolerance of plants to waterlogging stress [13]. However, excess accumulation of fermentation products (such as lactate, acetaldehyde, and ethanol) can cause problems in plant roots [14]. 

Amino acids are important substrates for the synthesis of nitrogenous substances, hormones, and secondary metabolites in plants [15]. Interconversion of amino acids is an important metabolic adjustment made by plants under waterlogging stress [16,17]. Unlike fermentation pathways, the metabolism of pyruvate to alanine provides a non-detrimental end product of anaerobic metabolism [18]. The quantity composition of amino acids is an important basis for the protein structure. In *Nicotiana tabacum*, the expression of glycine-rich RNA-binding protein-1 is upregulated under flooding stress [19]. It is important to unveil the biological significance of specific amino acids or classes of proteins rich in unique amino acids in terms of waterlogging tolerance in plants. 

Female kiwifruit scions are generally grafted on top of rootstocks, which are selected for desirable characteristics such as tolerance to waterlogging stress. Previously, *Actinidia valvata* plants have shown greater tolerances to waterlogging stress than *Actinidia deliciosa* [20,21]. *A. valvata* root systems can survive for a longer time, maintain relatively high activity, and alleviate oxidative stress under waterlogging stress [21,22]. However, the primary metabolism of *A. valvata* roots against waterlogging stress has not been well elucidated. Therefore, the present study was designed to compare responses of KR5 (rootstock from *A. valvata*) and ‘Hayward’ (rootstock from *A. deliciosa*) plants during waterlogging stress. Relative water content (RWC), gas exchange data, and sucrose content were measured in aboveground tissues of kiwifruit plants. For roots, we measured the sucrose content, fermentative enzyme activity, fermentation metabolite content, and amino acid content. Our results provided new information regarding the primary metabolism of *A. valvata* roots against waterlogging stress, which has not previously been well elucidated. 

## 2. Results

### 2.1. Relative Water Content, Malonaldehyde, and Gas Exchange Data under Waterlogging Stress

**Figure 1:**
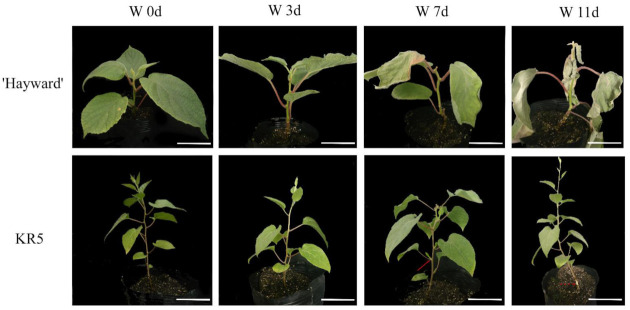
Morphological changes of ’Hayward’ and KR5 plants under waterlogging stress. W 0d, W 3d, W 7d, and W 11d indicate 0, 3, 7, and 11 d after waterlogging stress, respectively. ’Hayward’, from *Actinidia deliciosa*, and KR5 from *Actinidia valvata*. The solid and dashed arrow shows the newly formed sprout and callus in KR5 under waterlogging stress, respectively. Bar = 10 cm.

Wilting and drooping were first induced in ‘Hayward’ leaves after 7 d of waterlogging, and these symptoms were aggravated after 11 d of waterlogging (Figure 1). During the 11-day waterlogging process, KR5 leaves maintained health, as indicated by new sprouts and calluses that were observed in KR5 after 7 and 11 d of waterlogging, respectively (Figure 1). 

**Figure 2:**
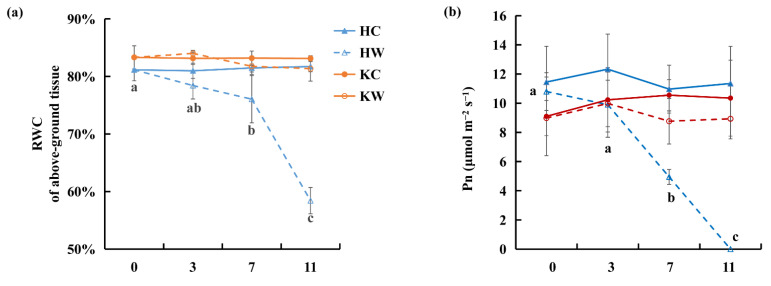

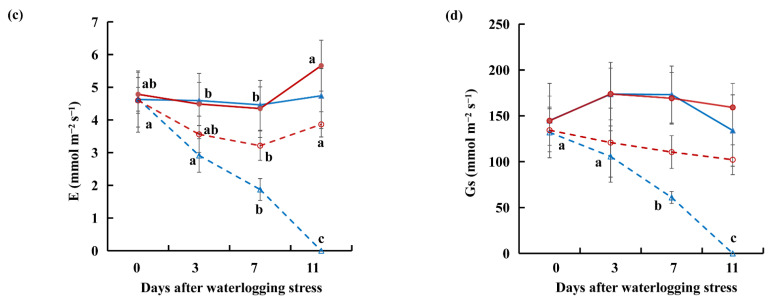

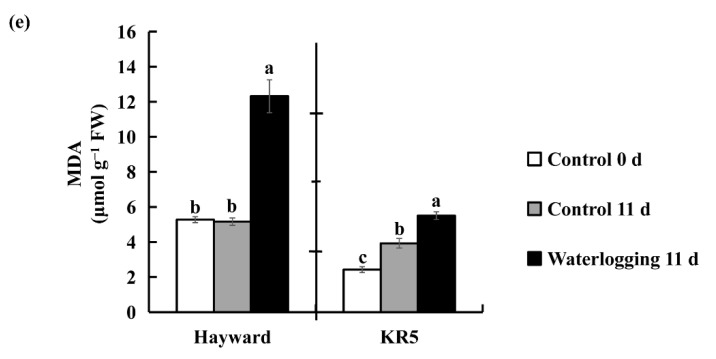
Relative water content (RWC), gas exchange data, and malondialdehyde (MDA) content of KR5 and ’Hayward’ plants under waterlogging stress. (**a**) RWC of aboveground tissue; (**b**) net photosynthetic rate, Pn; (**c**) transpiration rate, E; (**d**) stomatal conductance, Gs, and (**e**) MDA content of leaf. HC, control of ’Hayward’; HW, waterlogging of ’Hayward’; KC, control of KR5; KW, waterlogging of KR5; FW, fresh weight. Values are the mean ± SD (*n* = 6). Mean separations are compared statistically among different durations of waterlogging per genotype of rootstock per treatment by analysis of variance (ANOVA) at the 0.05 level of confidence, and significant differences are indicated by different letters (Fisher’s least significant difference test).

A stable RWC of aboveground tissue was maintained in both ‘Hayward’ and KR5 control plants, which were kept non-waterlogged (Figure 2a). For waterlogging treatments, the RWC of aboveground tissue of ‘Hayward’ was significantly lower than that of the 0-d control after 7 d of waterlogging (*p* = 0.02) and decreased to < 0.6 after 11 d of waterlogging (Figure 2a). In contrast, there was no significant decrease in the RWC of aboveground tissue of KR5 within 11 d of waterlogging stress at *p* < 0.05, and it remained > 0.8 after 11 d of waterlogging. 

More rapid decreases in the net photosynthetic rate (Pn), transpiration rate (E), and stomatal conductance (Gs) were induced in ‘Hayward’ leaves after 7 and 11 d of waterlogging stress, compared with those of KR5 (Figure 2b–d). After 7 d of waterlogging, the Pn of ‘Hayward’ plants were significantly less than that of the 0-d control (*p* = 4 × 10^−5^). The Pn of KR5 plants was maintained at a high level, similar to that of the 0-d control, after 7 d of waterlogging. Compared to the 0- or 11-d controls, the MDA content of leaves significantly increased after 11 d of waterlogging in both rootstocks at *p* < 0.05 (Figure 2e). However, the MDA content of ‘Hayward’ leaves was considerably higher than that of KR5 after 11 d of waterlogging. It can be concluded that KR5 leaves were less severely damaged and functioned more normally under waterlogging stress than ‘Hayward’ leaves. 

### 2.2. Morphological Changes of Stem Base and Root under Waterlogging Stress

**Figure 3:**
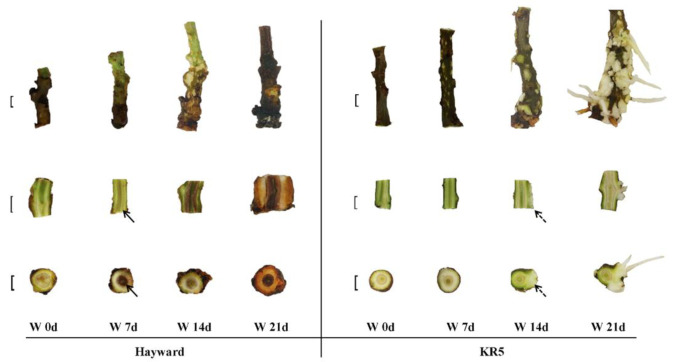
Morphological changes of ’Hayward’ and KR5 stem bases under waterlogging stress. W 0d, W 7d, W 14d, and W 21d indicate 0, 7, 14, and 21 d after waterlogging stress, respectively. Solid arrows show the early damage to tissues of ’Hayward’ after 7 d of waterlogging and dashed arrows show the newly formed calluses in KR5 after 14 d of waterlogging stress. Bar = 0.5 cm.

**Figure 4:**
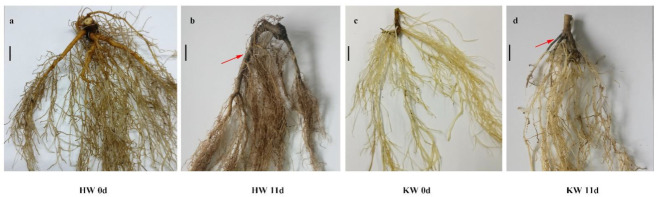
Morphological changes of roots of (**a**,**b**) ’Hayward’ and (**c**,**d**) KR5 under control and waterlogging conditions. HW 0d and HW 11d indicate ‘Hayward’ roots after 0 and 11 d of waterlogging stress, respectively. KW 0d and KW 11d indicate KR5 roots after 0 and 11 d of waterlogging stress, respectively. Arrows show the damaged root tissue, which turned black. Bar = 1 cm.

Longitudinal and cross-sections of the stem base showed that internal tissues started to turn brown in as few as 7 d of waterlogging and that the damage became more aggravated after the period extended to > 7 d in ’Hayward’ (Figure 3). On day 21 of waterlogging, stem bases were badly rotten without any formation of adventitious roots in ’Hayward’. For KR5, both longitudinal and cross-sections indicated that the internal tissues of stem bases maintained health during a 21-d waterlogging period. In addition, numerous hypertrophied lenticels were formed on the surface of stem bases after 7 d of waterlogging (Figure 3). Calluses and adventitious roots were gradually induced by prolonged waterlogging stress in KR5. The entire main root system of ’Hayward’ was severely damaged after 11 d of waterlogging, exhibiting a black color and high mortality (Figure 4). In contrast, only a small portion of feeder roots of KR5 decayed, and the majority of the main root system survived after 11 d of waterlogging (Figure 4). Overall, stem bases and roots of KR5 plants exhibited healthier appearances than those of ’Hayward’ under waterlogging stress.

### 2.3. Changes of Sucrose Content under Waterlogging Stress

**Figure 5:**
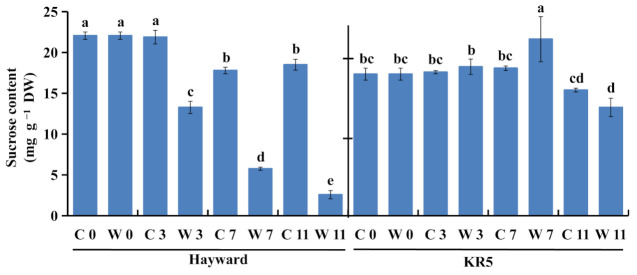
Sucrose contents of ‘Hayward’ and KR5 plants under control or waterlogging conditions. DW, dry weight. C 0, C 3, C 7, and C 11 indicate 0, 3, 7, and 11-d controls, respectively. W 0, W 3, W 7, and W 11 indicate waterlogging treatments of 0, 3, 7, and 11 d, respectively. Values are the mean ± SD (*n* = 3). Significant differences between treatments per genotype at *p* < 0.05 are indicated by different letters over columns (Duncan’s test).

After >3 d of waterlogging, sucrose content became significantly decreased in roots of ‘Hayward’ at *p* < 0.05, compared with corresponding controls (Figure 5). After 11 d of waterlogging, sucrose content was decreased to about 13.91% of that of the 11-d control. For KR5, sucrose content was increased within 7 d of waterlogging compared with corresponding controls (Figure 5). When waterlogging stress was extended to 11 d, sucrose content was decreased to about 86.17% of that of the 11-d control in KR5 roots. Probably, compared to ‘Hayward’, KR5 plants accumulating sucrose in roots to survive waterlogging stress was advantageous. 

### 2.4. Root Fermentations under Waterlogging Stress

**Figure 6:**
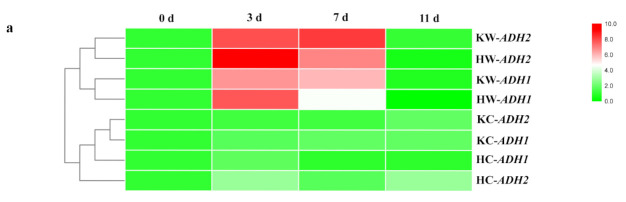

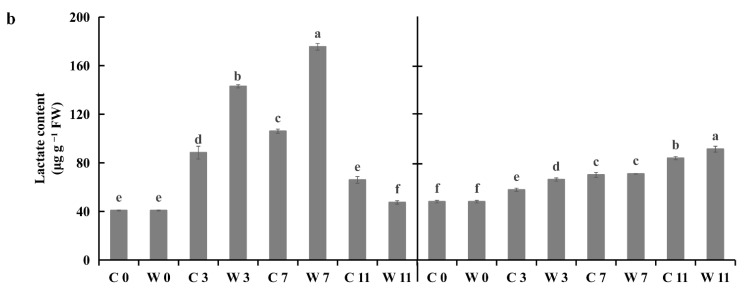

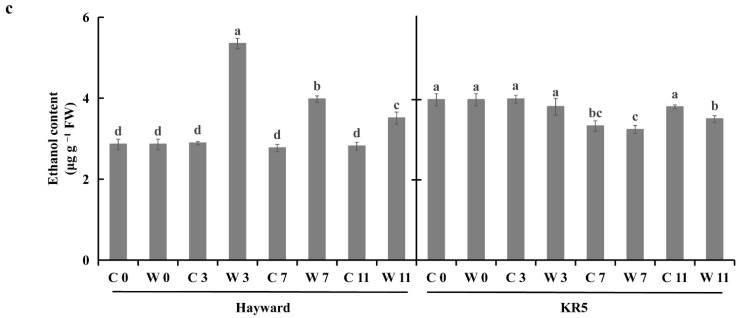
Gene expressions and metabolite contents in ‘Hayward’ and KR5 roots under control or waterlogging conditions. (**a**) Expression levels of *AdADH1* and *AdADH2*; (**b**) lactate contents; (**c**) ethanol contents. C 0, C 3, C 7, and C 11 indicate 0, 3, 7, and 11-d controls, respectively. W 0, W 3, W 7, and W 11 indicate waterlogging treatments of 0, 3, 7, and 11 d, respectively. Values are the mean ± SD (*n* = 3). Significant differences between treatments per genotype at *p* < 0.05 are indicated by different letters over columns (Duncan’s test).

ADH is a marker gene for hypoxic stress. Previously, two ADH genes (*AdADH1* and *AdADH2*) were reported to be significantly increased in the roots of *A. deliciosa* after 4 d of waterlogging treatments [23]. Similarly, in the present study, the expression of both ADHs was greatly induced in roots of both rootstocks within 7 d of waterlogging stress (Figure 6a). Lactate contents were significantly increased in ‘Hayward’ roots after 3 (*p* = 0.002) and 7 d (*p* = 3.8 × 10^−4^) of waterlogging (Figure 6b). For KR5, the lactate content also increased in roots under waterlogging stress but remained lower than that in ‘Hayward’. Notably, within 11 d of waterlogging, ethanol contents in waterlogged roots of ‘Hayward’ were maintained higher than that of their respective controls, whereas the opposite occurred in KR5 (Figure 6c). 

**Figure 7:**
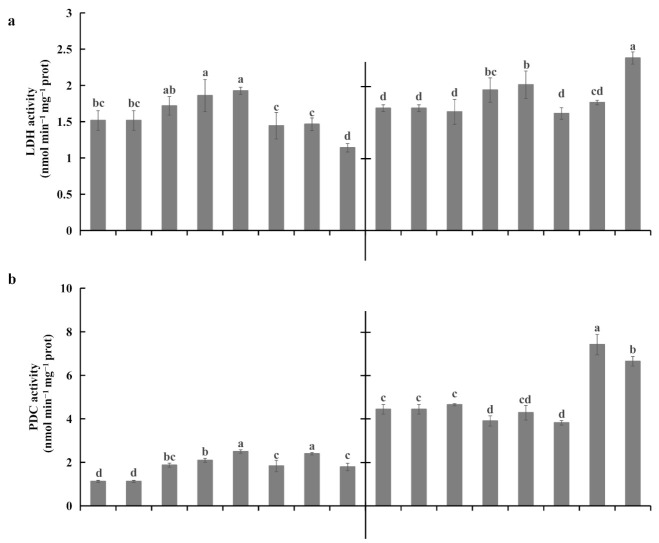

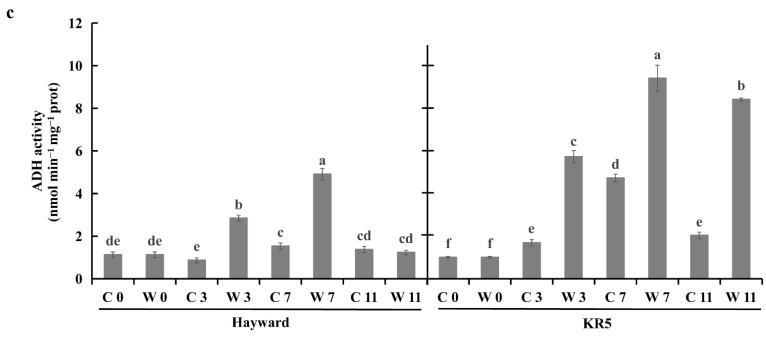
Fermentative enzyme activities in roots of ‘Hayward’ and KR5 under control and waterlogging conditions. (**a**) Lactate dehydrogenase (LDH) activity; (**b**) pyruvate decarboxylase (PDC) activity; and (**c**) alcohol dehydrogenase (ADH) activity. C 0, C 3, C 7, and C 11 indicate 0, 3, 7, and 11-d controls, respectively. W 0, W 3, W 7, and W 11 indicate waterlogging treatments of 0, 3, 7, and 11 d, respectively. Values are the mean ± SD (*n* = 3). Significant differences between treatments per genotype at *p* < 0.05 are indicated by different letters over columns (Duncan’s test).

Lactate dehydrogenase (LDH) activity increased after 3 d of waterlogging and declined after 7 d of waterlogging in both rootstocks (Figure 7a). However, after 11 d of waterlogging stress, LDH activity was significantly higher in KR5 roots than in controls (*p* = 0.004). After 3 d of waterlogging, PDC activity increased in ‘Hayward’ but decreased in KR5 (Figure 7b). Thereafter, both rootstocks responded to 7 and 11 d of waterlogging by decreasing PDC activity. PDC activity was higher in KR5 roots than in ‘Hayward’ roots under both control and waterlogging conditions. ADH activity was significantly promoted in the roots of both rootstocks within 7 d of waterlogging stress at *p* < 0.05 compared with respective controls (Figure 7c). However, after 11 d of waterlogging, ADH activity decreased to control levels in ‘Hayward’ roots, whereas it was still highly activated in KR5 roots. These results implied that KR5 plants were better able to maintain ethanolic fermentation and control the accumulation of detrimental anaerobic fermentation products in roots under waterlogging stress than ‘Hayward’ plants. 

### 2.5. Root Amino Acid Contents Under Waterlogging Stress

**Figure 8:**
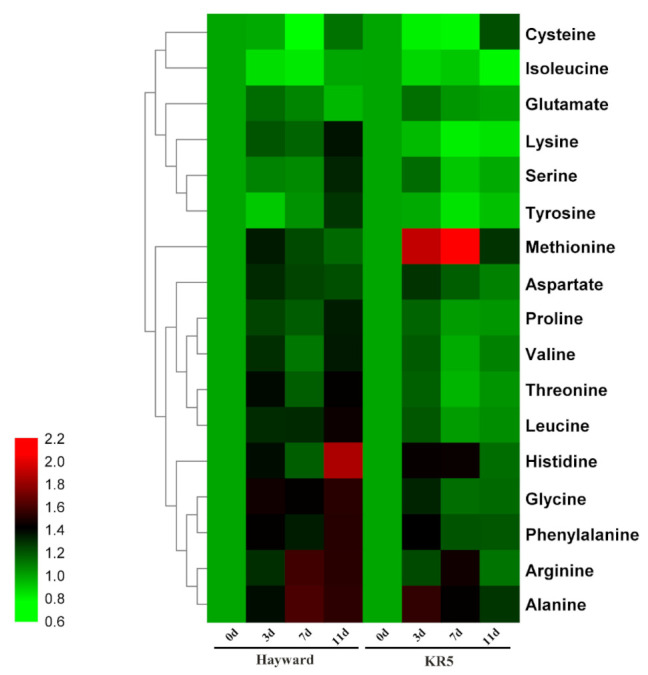
Change trends of individual amino acids in ‘Hayward’ and KR5 roots under waterlogging stress. For each amino acid, the value is computed by taking the ratio of the content of waterlogged plants and that of control plants.

**Figure 9:**
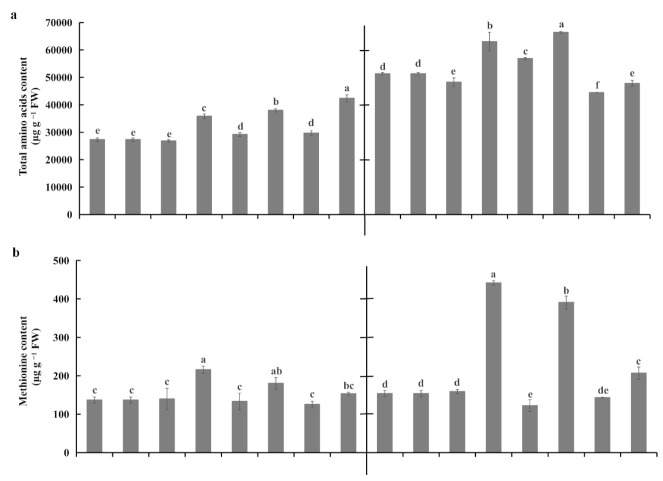

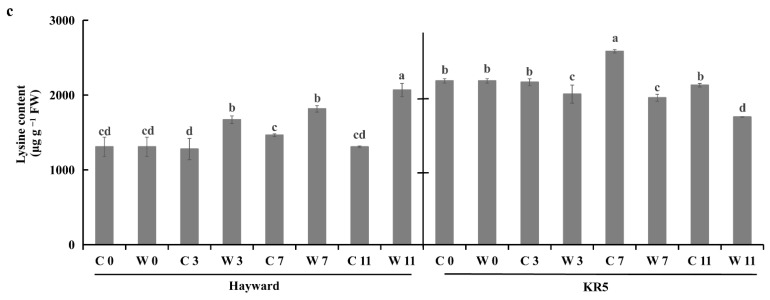
Concentrations of (**a**) total amino acids, (**b**) methionine, and (**c**) lysine in roots of ‘Hayward’ and KR5 under control and waterlogging conditions. C 0, C 3, C 7, and C 11 indicate 0, 3, 7, and 11-d controls, respectively. W 0, W 3, W 7, and W 11 indicate waterlogging treatments of 0, 3, 7, and 11 d, respectively. Values are the mean ± SD (*n* = 3). Significant differences between treatments per genotype at *p* < 0.05 are indicated by different letters over columns (Duncan’s test).

A heatmap analysis highlighted that the majority of tested amino acids accumulated more under waterlogging stress than under control conditions for both rootstocks (Figure 8). However, cysteine and isoleucine concentrations were simultaneously downregulated in the roots of both rootstocks under waterlogging stress (Figure 8). Waterlogging stress significantly enhanced the total amino acid concentration in roots of both rootstocks (Figure 9a). The total amino acid concentration was higher in KR5 than in ‘Hayward’ under both waterlogging and control conditions (Figure 9a). Compared with ‘Hayward’, methionine, arginine, and alanine contents were more remarkably increased in KR5 under waterlogging stress (Figure 8). For example, 1.55 and 2.78-fold changes in the methionine contents were induced in ‘Hayward’ and KR5 after 3 d of waterlogging, respectively (Figure 9b). Interestingly, compared with those in controls, lysine concentrations remained upregulated in ‘Hayward’ and down-regulated in KR5 under waterlogging stress (Figure 9c). It could be predicted that a higher level of total amino acids or specific adjustments of individual amino acids in roots rendered KR5 plants more adaptable to waterlogging stress than ‘Hayward’ plants.

### 2.6. Multivariate Data Analysis

**Figure 10:**
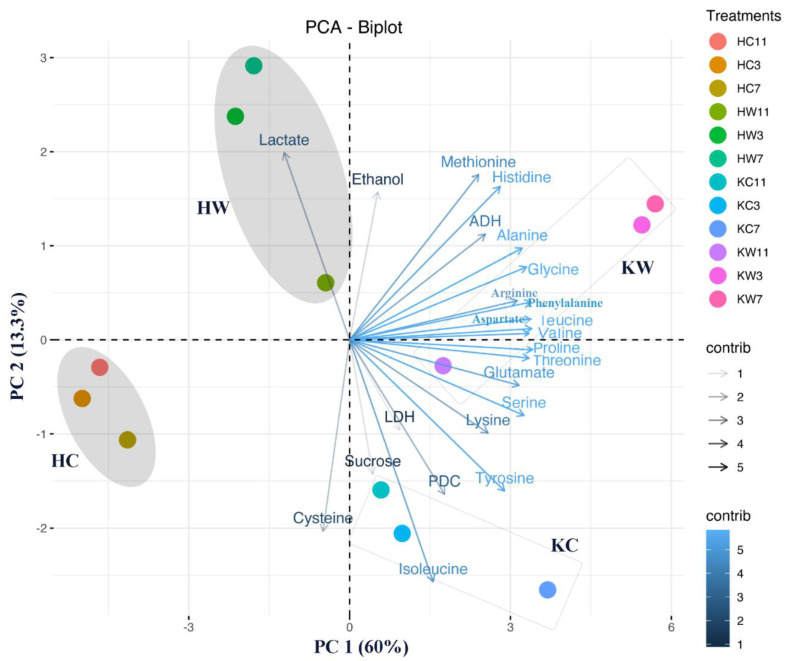
Bi-plot of the first and second principal components of evaluated root traits in ‘Hayward’ and KR5 under control and waterlogging conditions. HC, control of ‘Hayward’; HW, waterlogging of ‘Hayward’; KC, control of KR5; KW, waterlogging of KR5. Data from 3, 7, and 11d of waterlogging treatments and their respective controls are used for the analysis.

The principal component analysis (PCA) was performed for the dependent variables in two kiwifruit genotypes under different levels of waterlogging stress. The first two retained principal components (PCs) explained about 73.3% of the variance in the data (Figure 10). PC 1 had positive associations with all the individual amino acids (except cysteine), ADH and PDC. Meanwhile, this component was strongly negatively associated with lactate and cysteine levels. In PC 2, lactate, methionine, histidine, ethanol, ADH, and alanine had high positive loadings, whereas isoleucine, cysteine, PDC, tyrosine, and sucrose had high negative loadings. The distances between the control groups indicated the basic difference between these two rootstocks from different *Actinidia* species. Waterlogging treatments were further categorized into a KR5-waterlogging group and a ‘Hayward’-waterlogging group, which indicated the specific adjustments of each rootstock to waterlogging stress, as well as the effects of different waterlogging durations on the kiwifruit plants. Separate amino acids and ADH were directed to the KR5 waterlogging group. Lactate was clustered in the ‘Hayward’ waterlogging group.

## 3. Discussion

### 3.1. Kiwifruit Plant Growth under Waterlogging Stress 

Waterlogging stress initially causes injuries to the root, affecting the growth of the whole plant. Reduction in the Pn of leaves is a common response of plants to waterlogging stress [2,3]. Due to leaf dehydration, stomatal conductance usually declines under waterlogging stress. Stomatal closure was correlated with a decrease in the E and Pn under waterlogging stress [24]. In the present study, stomatal conductance was significantly decreased in ‘Hayward’ leaves under waterlogging stress, which was followed by a more rapid decrease in Pn. Leaf chlorosis is an indication of chlorophyll degradation, which also contributes to a decrease in Pn [25]. In the present study, a higher MDA content was induced in ‘Hayward’ leaves after 11 d of waterlogging. The noticeable decrease of Pn in ‘Hayward’ may have been due to severe photosystem damage. Overall, leaf performances well reflected the different waterlogging tolerances of KR5 and ‘Hayward’ plants. 

Morphological adaptations of stem bases have been previously observed in some flood-tolerant plants under flooding [26,27,28]. Development of adventitious roots can facilitate the entry of atmospheric oxygen into the stem and the efflux of toxins from roots [29,30]. In the present study, the formation of adventitious roots at the bases of the stem was an important anatomical acclimation for KR5 plants to adapt to waterlogging stress (Figure 3). A healthy status of the stem base is important for the formation of adventitious roots. For ‘Hayward’, no adventitious roots could be formed as their stem bases quickly rotted under waterlogging stress.

### 3.2. Root Sugar and Fermentative Metabolisms under Waterlogging Stress

The steady availability of carbohydrates in roots is crucial for maintaining glycolysis under waterlogging stress [31]. Soluble sugars also play a role in osmotic adjustment [32]. In this study, due to a lack of sucrose supply (Figure 5), ‘Hayward’ roots were more vulnerable than KR5 to the energy crisis after >3 d of waterlogging. Exogenous sucrose can greatly enhance the tolerance of Arabidopsis seedlings to anoxia [33]. Sucrose also functions as a signaling molecule that affects carbohydrate metabolisms and sucrose transport [34]. Our results exhibit that sucrose may play a role in the waterlogging tolerance of these two kiwifruit rootstocks. 

Our results show that excess accumulation of lactate will cause cytoplasmic acidification problems for ‘Hayward’ roots (Figure 6b). Arabidopsis plants are able to exude lactate outside the root, preventing it from accumulating to toxic levels in the cells [35]. Notably, compared with ‘Hayward’, KR5 roots accumulated less ethanol under waterlogging stress (Figure 6c). Exportation of root-derived ethanol to leaves has been reported in some plants under flooding [36,37]. Reusing the carbon exported from the roots is important for avoiding carbon loss problems under waterlogging stress.

PDC and ADH are two enzymes that play roles in the ethanol fermentation pathway. In the current study, compared with those in ‘Hayward’, activities of both enzymes were at higher levels in KR5 roots under waterlogging stress (Figure 7b,c), reflecting the higher tolerance of KR5 plants to waterlogging. PDC plays a regulatory role in ethanolic fermentation [38]. In the present study, continued down-regulation of PDC activity may have been linked to the sustained decrease in ethanol production in KR5 roots under waterlogging stress. Induction of transcript abundance of *ADHs* and enzyme activity are typical responses to hypoxic conditions in many plant species [31,39,40]. Activation of ADH activity promotes NAD^+^ regeneration and optimizes plant metabolism under hypoxic stress [41,42,43]. In this study, the drastic decline of ADH activity in ‘Hayward’ roots after 11 d of waterlogging stress may have affected the continuation of the glycolytic pathway.

### 3.3. Root Amino Acid Metabolisms under Waterlogging Stress

Amino acids play a role in plant stress adaptations by accumulating osmolytes, regulating cytoplasmic pH, and detoxifying reactive oxygen species [44]. In the present study, both rootstocks accumulated higher amounts of total amino acids in roots in response to waterlogging stress (Figure 9a). Both free and protein-bound amino acids contribute to elevated amino acid pools. The most pronounced upregulated changes were found for methionine, arginine, and alanine (Figure 8 and Figure 9b). Methionine tends to be oxidized under elevated reactive oxygen species levels [45]. The higher methionine content in KR5 roots was possibly related to its ability to scavenge reactive oxygen species upon waterlogging stress. Arginine is a precursor of polyamines, which can confer flooding tolerance to some plant species by influencing antioxidative systems or anaerobic respiration in roots [46,47]. Arginine-rich cationic proteins are known to stabilize the macromolecular structures [48]. In the present study, kiwifruit plants exhibited a common phenomenon of plants, accumulating a higher alanine content under waterlogging stress. Alanine accumulation can retain carbon and nitrogen resources and regulate the pH balance within anoxic cells [43,49]. Lysine specifically accumulated in ‘Hayward’ roots under waterlogging stress (Figure 9c). A high concentration of lysine was previously reported to be toxic to roots [50]. Metabolism of free lysine and lysine-rich proteins may influence the health of kiwifruit roots under waterlogging stress.

## 4. Materials and Methods

### 4.1. Plant Materials and Waterlogging Treatments

In this study, we used clonally propagated plants of KR5 (a waterlogging-tolerant rootstock from *A. valvata*) and ‘Hayward’ (a waterlogging-sensitive rootstock from *A. deliciosa*) as materials. ‘Hayward’ is a famous green kiwifruit cultivar; however, sometimes, their plants are used as rootstocks in kiwifruit production. Plantlets obtained from tissue culture were transferred to 18-cm-diameter pots containing a mixture of peat moss, perlite, and sand (1:1:1 *v/v*) and were managed in a greenhouse. The in-house environment was controlled at 65–75% relative humidity, 25–28 °C air temperature, and without shading. Three-month-old plants were used for the waterlogging treatments. To apply waterlogging stress, two potted plants (one pot of each genotype) were placed in a plastic container (45 × 35 × 16 cm) filled with tap water, and the water level was continuously maintained at 2–3 cm above the soil surface. Control plants were kept under the same conditions but without waterlogging. Waterlogged and control plants were randomly placed in a greenhouse. The waterlogging treatment periods were 0, 3, 7, and 11 d. Plant materials were harvested at the end of each time point of waterlogging treatments. Freshly harvested tissues were used to measure anaerobic fermentative enzyme activity. Some plant materials were frozen and stored at −80 °C for measurements of MDA, fermentative metabolite products, and amino acids, and for RNA extraction. Additionally, some plants from each rootstock were waterlogged for 14 and 21 d to monitor the morphological changes at the basal part of the stem. 

### 4.2. Measurements of Relative Water Content 

At each time point, six plants per genotype of rootstock per treatment were harvested, and the fresh weight (FW) of aboveground tissue was immediately measured. The RWC was measured by using a previously described method [51]. 

### 4.3. Gas Exchange Data and Measurement of Malondialdehyde Contents

The Pn, E, and Gs were measured using a portable photosynthesis system Li-6400 (Li-Cor, Inc., Lincoln, NE, USA) from 9:30 a.m. to 11:30 a.m., with a PLC3 universal/bryophyte leaf cuvette (18 × 25 mm). The area of the lamina that was exposed in the cuvette was approximately 4.5 cm^2^. Photosynthetically active radiation (PAR) was set at 1200 μmol m^−2^ s^−1^, according to a previous study [52]. Three fully expanded leaves from six marked plants per genotype of rootstock per treatment were used for continuous measurements of gas exchange data. MDA content was measured according to the procedure described in a previous study [53]. The MDA concentration was expressed as nmol per gram of FW.

### 4.4. Measurements of Sucrose Contents, Enzymatic Activities, and Metabolite Contents

Sucrose was measured by high-performance liquid chromatography according to a previous procedure [54]. Plant materials were oven-dried at 65 °C until their weights were constant. Then, ground samples were immersed in 80% ethanol and kept overnight. After centrifugation at 8000× *g* for 10 min, the extracts were passed through a syringe filter and then used for analysis. The chromatography separation was done by a Carbomix Ca-NP 8% column at 80 °C. In the mobile phase, water was used with an isocratic flow rate of 0.4 mL min^−1^. Sugars were detected with a differential refractometer (Waters 2414) and quantified using standards for sucrose.

Activities of LDH, PDC, and ADH were assayed using commercial kits (Suzhou Michy Biomedical Technology Co., Ltd., Suzhou, China). Approximately 0.1 g of plant material was homogenized in precooled extraction buffer on ice. After the mixture was centrifuged at 4 °C, the supernatant was used for the activity assay. LDH catalyzes the production of pyruvate by lactic acid, which reacts with 2,4-dinitrophenylhydrazine to form a red-brown complex in alkaline solution. LDH activity was indirectly assayed by monitoring pyruvate production rate. To assay for PDC, pyruvate was added to the reaction buffer containing ADH. PDC activity was assayed by monitoring the decrease in the absorbance of the reduced form of nicotinamide adenine dinucleotide (NADH) at 340 nm. Acetaldehyde was added to the reaction buffer of ADH to initiate the reaction. ADH activity was assayed by monitoring the rate of decrease in NADH at 340 nm. A bicinchoninic acid protein assay kit was used to measure the total soluble protein levels. One unit of LDH, PDC, and ADH was defined as the amount of enzyme required to decompose 1 nmol of substrate per milligram of protein per minute.

Ethanol content was analyzed using SP-3420a gas chromatography equipped with a head space BFRL autosampler and a KB-624 column. Approximately 1 g of ground sample was extracted using 2 mL ultrapure water in a glass bottle. After sealing, the bottle was placed aside for 20 min. The resulting gas was used for detecting the ethanol content according to a previously described procedure [55]. 

Quantitative analysis of lactate was performed with a RIGOL L-3000 high-performance liquid chromatography (HPLC) System according to a previously described procedure [56]. For sample preparation, 0.2 g of sample was immersed in 1.0 mL water and placed in an ultrasonic bath for 1 h, and then kept at 25 °C overnight. After centrifugation for 10 min at 8000× *g*, the supernatants were collected, adjusted to an equal volume (1.0 mL), and filtrated through a syringe filter for analysis. The separation was performed with a Kromasil reverse-phase C 18 column (250 × 4.6 mm, 5 μm). The column temperature was maintained at 30 °C, and eluent (sodium phosphate, 0.01 N, pH 2.8) was used at a flow rate of 0.8 mL min^−1^. Lactate was measured at a wavelength of 214 nm. 

### 4.5. Measurements of Amino Acid Contents

Amino acids (alanine, arginine, aspartate, cysteine, glutamate, glycine, histidine, isoleucine, leucine, methionine, phenylalanine, proline, serine, threonine, tyrosine, valine, and lysine) were measured simultaneously by a pre-column derivatization reverse-phase high-performance liquid chromatography method using phenyl isothiocyanate (PITC) [57]. For sample preparation, a 0.1 g of sample was mixed with 6 N hydrochloric acid and 0.1% phenol and transferred to a micro test tube after thorough homogenization. The tube was then placed in an oven for 20 h at 100 °C. After cooling to room temperature, 1 mL hydrolysate was collected and completely evaporated using a nitrogen-blowing instrument. The precipitate was dissolved in 1 mL of 0.1 N hydrochloric acid, and the solution used for derivatization was thoroughly filtered. Chromatographic analysis was carried out using a RIGOL L-3000 HPLC System. The Amethyst C18-H column (250 × 4.6, 5 μm) was maintained at 40 °C. The flow rate was set at 1.0 mL min^−1^, and the injection volume was 10 μL. The measurements were obtained at a wavelength of 254 nm. Mobile phase A: 7.6 g sodium acetate dissolved in 925 mL HPLC grade water (adjusted to pH 6.5 with acetic acid) +70 mL HPLC grade acetonitrile; mobile phase B: 80% HPLC grade acetonitrile mixed with HPLC grade water. The gradient conditions were as follows: initial conditions are 100% mobile phase A; from time 2 to 15 min, the gradient changes to 90% mobile phase A and 10% mobile phase B; from 15 to 25 min, the gradient changes to 70% mobile phase A and 30% mobile phase B; from 25 to 33 min, the gradient changes to 55% mobile phase A and 45% mobile phase B; from 33.1 to 38 min, the gradient changes to 100% mobile phase B; from 38.1 to 45 min, the gradient changes to 100% mobile phase A.

### 4.6. Gene Expression Analysis

Total RNA was extracted using a commercial RNA extraction kit (Huayueyang, Beijing, China). Total RNA was quantified using an Agilent 2100 Bioanalyzer (Agilent Technologies, Palo Alto, CA, USA) and NanoDrop (ThermoFisher Scientific Inc., Waltham, MA, USA), and 1% agarose gel. Approximately 1 μg of total RNA was used for reverse transcription, and complementary DNA (cDNA) was synthesized using a reverse Tra Ace qPCR RT kit according to the manufacturer’s protocol (TOYOBO, Osaka, Japan). Quantitative RT-PCR (qRT-PCR) reactions were performed using a LightCycler 480 II machine (Roche, Basel, Switzerland). A melting curve analysis was conducted to validate the amplification specificity of each run. The primer pairs for *act*, *AdADH1*, and *AdADH2* were selected from previous studies [23,58]. The kiwifruit *actin* gene (forward: 5′-TGCATGAGCGATCAAGTTTCAAG-3′, and reverse: 5′-TGTCCCATGTCTGGTTGATGACT-3′) was used as a normalizer. The *AdADH1* primer (forward: 5′-TGGAGTGTACTGGAAGTGTCAATG-3′, and reverse: 5′- AGCAGGGAGGTCGGAACG-3′) and *AdADH2* primers (forward: 5′-CTGAAATGACCAGCGGAGGAG-3′, and reverse: 5′-TGTCTTGAATACGGCATCTTTGTG-3′) were used to calculate the relative expression levels of genes. 

### 4.7. Statistics Analysis and Heatmap Illustration

Statistical analyses were conducted using SPSS Statistics (version 22.0). Gas exchange and MDA data were compared statistically among different durations of waterlogging per genotype of rootstock per treatment by analysis of variance (ANOVA) at a confidence level of 0.05 (Fisher’s least significant difference test). For two waterlogging levels (i.e., waterlogging and non-waterlogging) and four time periods (0, 3, 7, and 11 d), the data were first evaluated by two-way ANOVA. When the F test was significant, the mean separations were determined using an ANOVA Duncan test (*p* < 0.05). PCA was performed using the FactoMineR package [59], and the results were extracted and visualized using the factoextra package. In detail, data was loaded by *decathlon2*; PCA function was conducted by *FactoMineR::PCA*, and results were visualized by *factoextra::fviz_pca*. A heatmap was generated using the software TBtools (a toolkit for biologists integrating various biological data-handling tools) [60], and graphic features were manually adjusted. 

## 5. Conclusions

Kiwifruit plants from KR5 (*A. valvata*) were much more tolerant to waterlogging stress than those from ‘Hayward’ (*A. deliciosa*) as determined by their healthy appearance, normal photosynthesis, and rapid formation of adventitious roots at the stem base. For KR5, metabolic adjustments such as sufficient sucrose reserves, regulation of fermentative enzymes, avoidance of excess lactate and ethanol accumulation, and promoted accumulation of total amino acids most likely contributed to the healthy root system status under waterlogging stress. Compared with ‘Hayward’, the higher tolerance of KR5 plants to waterlogging stress depended on both metabolic and morphological adaptations of roots. The different waterlogging tolerance ability of these two rootstocks is possibly due to the differences in sucrose and lysine metabolisms in roots. Only some important enzymes and metabolites were measured in this study; the ways in which carbohydrate and amino acids metabolisms are simultaneously operated in kiwifruit roots under waterlogging stress requires further explorations in the future.

## Data Availability

All data generated or analyzed during this study are included in this published article.
